# Social Determinants of Health and Biological Age among Diverse U.S. Adults, NHANES 2011–2018

**DOI:** 10.21203/rs.3.rs-4540892/v1

**Published:** 2024-06-24

**Authors:** Robert Mesa, Maria Llabre, David Lee, Tatjana Rundek, Katrina Kezios, Adina Zeki Al Hazzouri, Tali Elfassy

**Affiliations:** University of Miami Miller School of Medicine; University of Miami; University of Miami Miller School of Medicine; University of Miami Miller School of Medicine; Columbia University; Columbia University; University of Miami Miller School of Medicine

**Keywords:** Health equity, Accelerated aging, Racial/ethnic minority populations, Healthy aging

## Abstract

We examined the sex-specific association between education and income with biological age (BA) and by race/ethnicity. The Klemera-Doubal method was used to calculate BA among 6,213 females and 5,938 males aged 30–75 years who were Hispanic, non-Hispanic (NH) White, NH Black (NHB), or NH Asian (NHA). Compared with a college education, less than a high school education was associated with greater BA by 3.06 years (95% CI: 1.58, 4.54) among females only; associations were strongest among NHB, Hispanic, and NHA females. Compared with an annual income of ≥$75,000, an income <$25,000 was associated with greater BA by 4.95 years (95% CI: 3.42, 6.48) among males and 2.76 years among females (95% CI: 1.51, 4.01); associations were strongest among NHW and NHA adults, and Hispanic males. Targeting upstream sources of structural disadvantage among racial/ethnic minority groups, in conjunction with improvements in income and education, may promote healthy aging in these populations.

## Introduction

Social determinants of health (SDoHs) are fundamental drivers of health inequities.^1^ People of low socioeconomic status (SES) are burdened by premature morbidity and mortality^2,3^ and have an accelerated decline in physical, physiological, and cognitive health.^4^ Metrics of individual SES, such as education and income, are inversely associated with accelerated aging.^5,6^ This is particularly salient for racial and ethnic minority populations who, owing to structural disadvantage,^7^ disproportionately are of lower SES,^8^ and exposed to psychosocial stressors such as stigma and discrimination.^9^ Persistent stress from life-course social disadvantage has been posited to result in earlier aging^10^ and exacerbate health disparities among racial and ethnic minority populations.^11^ In fact, aging speed differs by race/ethnicity, with non-Hispanic Black (NHB) adults aging at a faster pace compared with non-Hispanic White (NHW) adults^12^ and Hispanics adults.^13^

The impact of SES on health and aging is not homogenous across race/ethnic groups. For example, the “diminishing returns hypothesis” posits that as SES levels increase, NHB adults do not have an equivalent improvement in health compared to NHW adults.^14^ Less is known about the association between SDoHs and aging speed among Hispanic and non-Hispanic Asian (NHA) adults, groups that represent fast-growing segments of the U.S. population.^15,16^ By 2060, ethnic and racial minorities will represent the majority of the U.S. population.^15,16^ Understanding the drivers of accelerated aging in traditionally understudied and underserved groups is critical for achieving health equity.

Chronological age (CA) refers to the amount of time passed since birth, while biological age (BA) refers to the phenotypic changes associated with the gradual aging process across the lifespan.^17^ Contemporary research in aging has emphasized the utilization of BA instead of CA as a marker of the body’s degradation and breakdown.^18^ Among the several algorithms used in the calculation of BA, the Klemera-Doubal Method (KDM) is regarded as the most valid.^19^ The overarching goals of this study were to describe differences in aging speed by SDoHs and race/ethnicity, determine whether SDoHs are associated with BA, and examine whether this association is modified by race/ethnicity.

## Results

Among males, mean CA was 50.4 years, 38.8% were NHW, 21.4% NHB, 23.0% Hispanic, and 12.9% NHA adults (Table 1). More than a third were college graduates (34.5%), 47.4% had an annual household income ≥ $75,000, and 82.6% were U.S. born. NHB and Hispanic males were more likely to be younger and of lower SES (*P* < 0.05). Among females, mean CA was 51.0 years, 36.9% were NHW, 22.0% NHB, 24.4% Hispanic, and 13.5% NHA adults (Table 2). More than a third were college graduates (34.6%), 41.8% had an annual household income ≥ $75,000, and 82.6% were U.S. born. NHB, Hispanic, and NHA females tended to be younger (30–44 years) and have less than a high school (HS) education, while NHB and Hispanic females had a lower household income (*P* < 0.05).

### BA and Aging Difference by sex, race/ethnicity, and SDoHs

**Figure 1:**
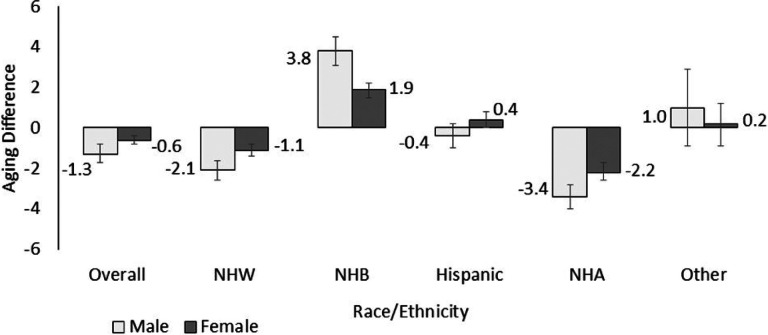
Aging difference **among males and females** and stratified by race/ethnicity.

Among males: mean BA was 49.1 or 1.3 years (95% CI: −1.7, −0.8) lower than CA (Figure 1). By race/ethnicity, aging difference was highest in NHB (3.8 years, 95% CI: 3.1, 4.5) and lowest in NHW (−2.1 years, 95% CI: −2.6, −1.6) and NHA males (−3.4 years, 95% CI: −4.0, −2.8). Among females: mean BA was 50.4 or 0.6 years (95% CI: −0.8, −0.4) lower than CA. By race/ethnicity, aging difference was highest in NHB (1.9 years, 95% CI: 1.5, 2.2) and lowest in NHW (−1.1 years, 95% CI: −1.4, −0.8) and NHA females (−2.2 years, 95% CI: −2.6, −1.7). Mean BA and CA by sex and race/ethnicity are shown in **Figure S2. Figure S3** shows NHB males and females had accelerated aging, while NHW and NHA males and females had decelerated aging across all levels of education and income. **Figure S4** shows that among immigrants, Hispanic males and NHA males and females had decelerated aging, while among those U.S. born, NHB and Hispanic males and females had accelerated aging.

### Associations between SDoHs and BA

Among males, from fully adjusted models (Table 3, model 3), an income <$25K compared to ≥$75K was associated with greater BA by 4.95 years (95% CI: 3.42, 6.48). Compared to U.S. born males, BA was 2.46 years lower (95% CI: −3.54, −1.37) among males who had lived ≥10 years in the U.S., and 2.78 years lower (95% CI: −4.12, −1.44) among males who had lived <10 years in the U.S. (model 4). Associations between educational attainment and BA were not significant (*P* >0.05) among males. Among females, compared to college graduates, BA was greater by 3.11 years among females who were HS graduates (95% CI: 2.00, 4.22) and greater by 3.06 years (95% CI: 1.58, 4.54) among females with less than a HS education. Compared to females with an income ≥$75K, BA was greater by 2.76 years among females with an income <$25K (95% CI: 1.51, 4.01). Compared to U.S. born females, BA was 1.30 years lower (95% CI: −1.85, −0.74) among females who had lived 10 years in the U.S., and 1.52 years lower (95% CI: −2.54, −0.51) among females who had lived <10 years in the U.S.

### Associations between SDoHs and BA according to race/ethnicity

**Figure 2:**
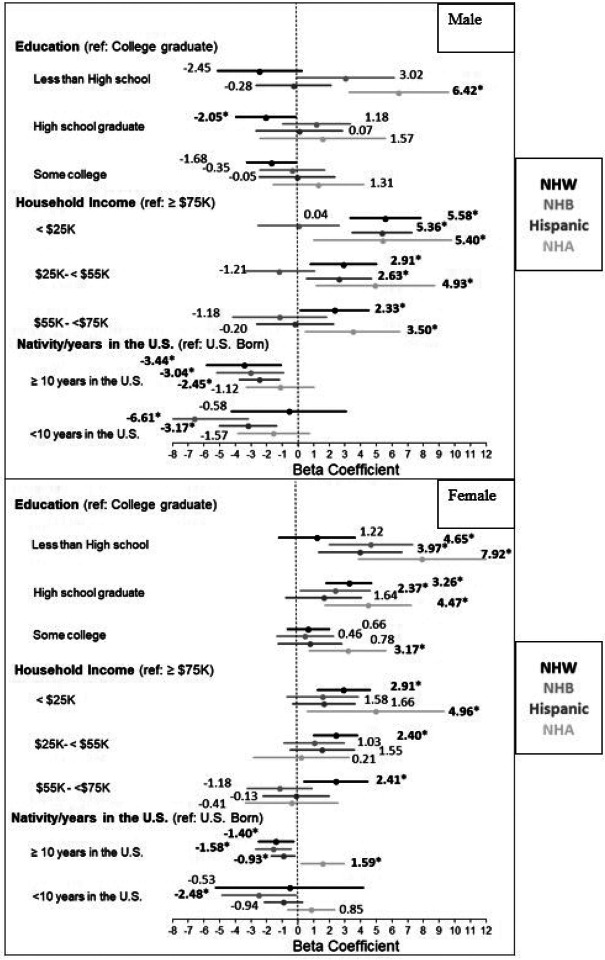
Forest Plot showing multivariable adjusted associations between SDoHs and Biological Age **by sex**, stratified by race/ethnicity. (Fully adjusted models) Model 3: adjusted for health insurance, marital status, education (in income and nativity models), income (in education and nativity models), nativity (in education and income models), alcohol use, smoking history, physical activity, and history of CVD Model 4: adjusted for Model 3 variables, and chronological age [Nativity/years residing in the U.S. models only]

Associations between all SDoHs (education, income, nativity/years residing in the U.S.) and BA significantly differed by race/ethnicity (*P* for interaction < 0.10) (Figure 2). Among males, compared to college graduates, less than a HS education was associated with greater BA by 6.42 years among NHA males only (95% CI: 3.25, 9.25). Compared to an income ≥$75K, an income <$25K was associated with greater BA by 5.58 years among NHW males (95% CI: 3.33, 7.83), 5.36 years among Hispanic males (95% CI: 3.47, 7.25), and 5.40 years (95% CI: 0.99, 9.81) among NHA males. Compared to being U.S. born, living in the U.S. <10 years was associated with lower BA by 6.61 years among NHB males (95% CI: −10.01, −3.17) and by 3.17 years (95% CI: −4.99, −1.36) among Hispanic males. Among females, compared to college graduates, less than a HS education was associated with greater BA by 4.65 years among NHB females (95% CI: 2.01, 7.29), 3.97 years among Hispanic females (95% CI: 1.34, 6.61), and 7.92 years among NHA females (95% CI: 3.87, 11.97). Compared to an income ≥$75K, an income <$25K was associated with greater BA by 2.91 years among NHW females (95% CI: 1.24, 4.59) and 4.96 years among NHA females (95% CI: 0.59, 9.33). Compared to being U.S. born, living in the U.S. <10 years was associated with lower BA by 2.48 years (95% CI: −4.82, −0.14) among NHB females.

### Sensitivity Analyses

Results of our sensitivity analysis using sex-independent BA were consistent with our main results showing that SDoHs were associated with BA among males and females (**Table S1**), and that race/ethnicity modifies the association (**Figure S5**).

## Discussion

In a nationally representative study of U.S. adults, we found stark differences in BA and aging difference by SDoHs and race/ethnicity. Aging difference was lowest (most favorable) among NHA adults and highest (least favorable) among NHB adults. Likewise, aging difference was higher among NHB adults compared to all other race/ethnic groups, across all levels of education and income (Supplemental Figure 3). In multivariable models, lower income, less educational attainment and more time spent in the U.S. were associated with greater BA, with differences by race/ethnicity. Common measures of SDoHs (education, income, and nativity/years residing in the U.S.), as well with the intersection of race/ethnic background, play an important role in aging.

Black-White differences in biological aging have been demonstrated by many prior studies.^10,12,20,21^ Results from the Coronary Artery Risk Development in Young Adults study, showed that by age 45, Black adults were on average 10 years older than their CA, while White adults were roughly 1.5 years younger.^20^ Likewise, findings from the Health and Retirement Study showed that NHB males were about 2 years and NHB females were 1 year older than their CA, while NHW males and females were roughly 1 year younger than their CA.^21^ Using the KDM method, we found that on average, NHB males and females were 3.8 and 1.9 years older, respectively, while NHW males and females were 2.1 years and 1.1 years younger, respectively, than their CA (Figure 1). Among Hispanic adults, research on differences between BA and CA have been mixed. Though, some studies point to accelerated aging among Hispanic adults,^13,21^ we found no significant difference between BA and CA among Hispanic persons. The “Hispanic Paradox” posits that despite an unfavorable socio-economic and biological risk profile, Hispanic adults have equivalent or better health outcomes and a longer life expectancy compared to NHW adults.^22^ Our results are consistent with this phenomenon, as we did not find any indication of accelerated aging among Hispanic people, despite relative socio-economic disadvantage in this population. Less is known about BA among Asian American populations. To date, most research on aging in Asian people has been conducted in mainland China. Research in Chinese adults suggests slower biological aging,^23^ with one study showing no difference between BA and CA.^24^ In our study, one of the few to include Asian Americans, we found NHA males were 3.4 years younger and NHA females were 2.2 years younger than their CA. However, we emphasize that these results should be interpreted with caution. Asian Americans are a heterogeneous population. At the national level, aggregated Asian Americans have favorable socio-economic profiles which may explain our findings.^25^ This is in contrast to regional or localized data from smaller studies of Asian sub-groups showing adverse socio-economic conditions and health disparities.^26^

Socio-economic disadvantage has been linked to accelerated aging.^5,6,21^ Findings from the Healthy Aging in Neighborhoods of Diversity Across the Life Span study found that income below the poverty level was associated with accelerated aging among Black and White adults.^27^ Similarly, we found the lowest compared to the highest income level was associated with a greater BA by five years in NHW, Hispanic, and NHA males, and by three and five years among NHW and NHA females, respectively (Figure 2). Education, an important SDoH, has also been implicated in the aging process. Among participants of the Multi-Ethnic Study of Atherosclerosis, lower education was associated with accelerated aging.^6^ Interestingly, in our study, lower educational attainment was associated with greater BA among females only (Table 3). Associations between SDoHs and health outcomes have previously been described among females in the literature.^28^ A potential explanation for different associations by sex may be attributed to differences in reactivity to socio-economic stressors. For example, in the Midlife in the United States study, lower SES was associated with a larger increase in negative emotions among females but not males, and this mediated the association between SES and physical health.^29^ In fact, research from the Health and Retirement study has shown that education is a more potent predictor of behavior changes in females.^30^ Therefore, compared to males, females with lower levels of education may be more vulnerable to unhealthier lifestyle choices^31^ and prone to stress and depression, which negatively impact their health^32^ and can contribute to advanced biological aging.

The ‘Weathering Hypothesis’ attributes the earlier manifestation of disease experienced by ethnic minority populations, and in particular NHB adults, to the cumulative effect of a lifetime of exposure to social or economic adversity.^10^ Persistent coping from stressors related to stigmatization and discrimination leads to unhealthy behaviors^33^ and exacerbates physiological deterioration.^10^ Consequently, despite SES improvement, NHB adults may not experience a net benefit in health. This “diminishing returns hypothesis” is evident in our study, as we found accelerated aging among NHB adults across all levels of education and income (Supplemental Figure 3). In contrast, all other race/ethnic groups showed signs of less aging with greater education and income attained. Despite the protective effect of high SES on healthy aging,^34^ our results are consistent with weathering among NHB males and females. A possible explanation for this association is that NHB adults of higher SES experience greater discrimination (which is not reflected through SES metrics), whether racial, gender, or lifetime, compared to other groups, including even those of lower SES.^35^

Immigrant health advantages have long been reported in the U.S., with foreign-born adults on average with a life expectancy 3.4 years longer compared with the native-born population.^36^ Consistent with prior literature,^21^ in our study, we found lower BA among adults who were foreign-born compared to their U.S. born counterparts (Table 3). Among immigrants, we found a shorter duration of U.S. residence was associated with slower aging, especially among racial/ethnic minority populations (Table 3, Figure 2). Acculturation, or the process by which one adopts the cultural patterns of the host country,^37^ has been linked with unhealthier lifestyle choices^38^ and dysregulation of biological stress markers, a precursor to adverse health outcomes,^39^ which may explain our findings and others. For example, the attenuation of immigrant health advantages with longer duration of time spent in the U.S. has been documented.^40^ In a study of diverse older adults, foreign-born Hispanics exhibited slower aging compared to U.S. born Hispanics.^21^ Among NHA adults, longer duration of U.S. residence is associated with greater consumption of ultra-processed food, a risk factor for adverse cardiometabolic health.^41^ Our results have population-wide implications. As the Hispanic population has shifted from predominantly an immigrant makeup to a majority U.S. born^16^ and the NHA population acculturates,^42^ effort is needed to help these groups retain the immigrant health advantage.

The current study is not without limitations. First, the cross-sectional design of this study limits our ability to assess life-course improvements in SES. Further, NHANES aggregates racial/ethnic populations, thereby masking sub-group differences. For example, previous research among Hispanic^43^ and NHA^44^ adults have found differential associations in health outcomes by background group. As previous research in aging in Hispanics has been in mainly Mexican populations, future research should assess the role of SDoHs with BA among disaggregated diverse adults, particularly of Hispanic or Asian descent. Additionally, an increasing concern with national surveys has been the declining response rate in contemporary survey cycles. Likewise, recent immigrants, who may be healthier compared to those U.S. born, may decline to participate because of language barriers.^45^ Finally, not all potential biomarkers were available across all NHANES data cycles. It is possible that including biomarkers not readily available, such as C-reactive protein and forced expiratory volume, would have improved BA estimation given the relationship between inflammation and lung function with aging.^18,46^ As BA indices were derived in a sex-specific manner and different biomarkers were retained among males and females, we were unable to test for statistical interactions by sex. However, in our sensitivity analyses, which utilized a sex-independent BA, we did find statistically significant interactions by sex, suggesting that our observed differences by sex persist despite our use of sex-specific BA algorithims. This study also has notable strengths. To the best of our knowledge, this study is the first to characterize BA among a representative and contemporary sample of U.S. Hispanic and NHA adults. In addition, we examine the role of several SDoHs (education, income, and nativity/years residing in the U.S.) in relation to BA among racial/ethnic minority populations. Further, we leveraged a novel approach in BA estimation and utilized the KDM in all our analyses.

In summary, in a representative sample of diverse U.S. adults we found evidence of accelerated aging among NHB adults compared to all other race/ethnic groups across all levels of education and income. We also found significant associations between SDoHs (education, income, and nativity/years in the U.S.) with BA. We found evidence of a protective effect of foreign-born nativity on BA among females and males, with an attenuation of the health advantage with a longer duration of residency in the U.S. These findings demonstrate that adverse levels of SDoHs are associated with premature biological aging and may have long-term implications for health outcomes. Further, given the accelerated aging among NHB adults even at higher levels of income or education, targeting upstream sources of structural disadvantage among racial/ethnic minority groups, in conjunction with improvements to income and education, are likely required to help achieve health equity in aging.

## Methods

### Study Population

The National Health and Nutrition Examination Survey (NHANES) is an ongoing, cross-sectional survey, that is representative of the non-institutionalized U.S. civilian population. The NHANES has been conducted in two-year cycles continuously since 1999. Asian Americans were oversampled beginning with the 2011–2012 data cycle. Data for this study were sourced from four NHANES cycles (2011–2018). Trained interviewers collected demographic, socioeconomic, dietary, and health-related information in the participant’s home. Participants attended a mobile examination center (MEC) where physical examinations and laboratory testing were conducted under standardized protocols. NHANES protocols were approved by the Ethics Review Board of the National Center for Health Statistics and all methods were performed in accordance with the relevant guidelines and regulations. Written informed consent was obtained prior to data collection.

### Measures

#### Social Determinants of Health (SDoHs)

Education, household income, and nativity/years residing in the U.S. were obtained from self-reported questionnaires. Education: was measured from participants’ highest educational level attained at the time of interview (categorized as: less than high school, high school/GED equivalent, some college or an Associate degree, or a college graduate and above). Household income: was measured from participants total annual household income (categorized as: <$25K, $25K-<$55K, $55K-<$75K, or $75K+). Nativity/years residing in the U.S.: was measured from participants country of birth in combination with reported length of time spent in the U.S. Participants were categorized as U.S. born, foreign born and residing in the U.S. for ≥ 10 years, or foreign born and residing in the U.S. < 10 years.

#### Biological Age (BA)

BA was calculated using the KDM.^47^ The KDM is a multi-step process that involves: 1) the identification of biomarkers to be used in the KDM algorithm, 2) the utilization of selected biomarkers in a reference population, and 3) the application of the reference training parameters in the analytic dataset. BA is then computed using an algorithm that utilizes the parameter values derived from the reference population and biomarker values from the analytic sample.^48^ Each step is outlined in further detail below.

##### Identification of biomarkers:

We first identified biomarkers that are associated with aging^46^ and were consistently collected across all NHANES data cycles. The following 15 biomarkers were considered: Blood Urea Nitrogen (BUN), Serum Creatinine, Albumin, Alanine Aminotransferase (ALT), White Blood Cell (WBC) Count, Total Cholesterol (TC), High-Density Lipoprotein Cholesterol (HDL), non-HDL cholesterol (TC minus HDL cholesterol), Glycosylated Hemoglobin (HbA1c), Waist Circumference (WC), Body Mass Index, Systolic Blood Pressure (SBP), Pulse Pressure (PP, the difference between systolic and diastolic blood pressure), Alkaline Phosphatase (ALP), and Albumin-to-creatinine ratio (ACR). The biomarkers identified account for the aging process in different organ systems.^46^

##### Selection of biomarkers:

In order to be independent of BA estimation in the analytic dataset, we derived KDM-BA algorithm parameters using NHANES data cycles 2007–2008 and 2009–2010 as the reference population.^49^ We derived models separately for males and females, as biomarker distributions differ by sex.^50^ We used correlation analyses to obtain sex-specific Pearson correlation coefficients between each biomarker with CA. We selected significantly correlated biomarkers with CA at *r* > 0.10^48^ and used a threshold of *r* > 0.7 to determine multi-collinearity between the biomarkers. From the initial list of 15 biomarkers, nine were selected in males and females. Among males, the following were retained: BUN, serum creatinine, albumin, ALT, non-HDL cholesterol, HbA1c, WC, PP, and ACR. Among females, the following were retained: BUN, serum creatinine, WBC, non-HDL cholesterol, HbA1c, WC, PP, ALP, and ACR. We re-ran the correlation analysis after removing missing data from the nine biomarkers, and excluded biomarker values that were more than five standard deviations away from the sex-specific mean.^49^ Each significantly correlated biomarker was then individually regressed on CA to obtain the intercept, slope, and root mean squared error (RMSE).^47^ A final set of parameters contained the intercept, slope, RMSE, and correlation coefficients.

##### Application:

Described in detail elsewhere,^48,49^ in Step 1, we calculated an initial estimate of BA using an equation containing biomarker values from the analytic sample, and the intercept, slope, and RMSE values from the reference dataset. In Step 2, we calculated an overall correlation coefficient using the significant correlation coefficients obtained in the reference dataset. In Step 3, we calculated the variance in BA using the initial BA estimate from Step 1, the overall correlation coefficient from Step 2, and each individual CA. In Step 4, we obtained a robust estimate of BA using a modified Step 1 equation that included the scaling variance factor derived in Step 3. BA was calculated for all males and females, separately. As a secondary outcome, to characterize aging speed, we calculated aging difference, defined as the difference between BA and CA. A positive aging difference was indicative of accelerated aging, while a negative aging difference was indicative of decelerated aging.

As an alternative measure, we also estimated sex-independent BA in the whole sample (e.g. in males and females as one group). Eight biomarkers were retained after the correlation analyses for this secondary measure: BUN, serum creatinine, albumin, HbA1c, WC, SBP, ALP, and ACR. Subsequent steps were followed in the same manner.

#### Other Variables of Interest

Participants self-reported demographic and health behaviors via standardized questionnaires. Participants reported race and Hispanic origin and identified as non-Hispanic White, non-Hispanic Black, Hispanic, non-Hispanic Asian, or another race (which includes multi-racial). Participants also disclosed marital status (married or living with a partner, or never married/widowed/divorced/separated), health insurance status (yes/no), alcohol use (yes/no on consumption of at least 12 alcoholic beverages in the past year), smoking status (current smoker, former smoker, or never smoker), and history of cardiovascular disease (CVD) (self-reported history of congestive heart failure, coronary heart disease, angina/angina pectoris, a heart attack, or a stroke). Physical activity was measured from the physical activity questionnaire (based off the Global Physical Activity Questionnaire). Participants self-reported whether they engaged in vigorous intensity (i.e., running or playing basketball) or moderate intensity (i.e., brisk walking, swimming, or golf) recreational activities for at least 10 minutes continuously in a typical week, or no recreational activity.

#### Analytic Sample

Of the 39,156 adult and child participants from NHANES 2011–2018 cycles, we excluded n=15,331 children (<18 years of age) and n=7,178 adults outside of the inclusionary age range (30–75 year olds were included to ensure biomarkers reflected age-related variation and to minimize survival bias by not including people with better than average longevity). We also excluded participants missing information on all three SDoHs (education, income, or nativity/years residing in the U.S.) (n=1,916). Given that laboratory values vary during pregnancy, we excluded pregnant females^10^ (n=94) and participants without complete information on all biomarkers utilized in the KDM calculation (n=2,486). The final analytic sample included 12,151 adults (5,938 males and 6,213 females). A study flow chart is shown in **Figure S1**.

#### Statistical Analysis

We described population characteristics (age group, nativity/years residing in the U.S., education, income, health insurance, marital status, alcohol use, smoking history, physical activity level, and history of CVD) by sex and stratified by race/ethnicity. We determined whether characteristics differed by race/ethnicity using ANOVA for continuous variables or chi-square tests for proportions. For males and females, we estimated mean aging difference by race/ethnicity and SDoHs. Next, we used multivariable linear regression models to determine the association between education, income, and nativity/years residing in the U.S. with BA. Model 1 was adjusted for race/ethnicity. Model 2 included Model 1 covariates in addition to education, income, health insurance, marital status, and nativity/years residing in the U.S. Model 3 included Model 2 covariates in addition to alcohol use, smoking history, physical activity level, and history of CVD. We considered Model 3 to be our fully adjusted model. We also included an additional Model 4 which included Model 3 covariates and CA. To determine whether race/ethnicity modified the association between SDoHs and BA, we tested the multiplicative interaction between each SDoH and race/ethnicity. Finally, in order to facilitate sex-based comparisons of our results, in a sensitivity analysis, we repeated our multivariable linear regression models using our alternative (sex-independent) BA variable as an outcome. We did so, as statistically comparing results across males and females was not possible with sex-specific calculation of BA (our main BA measure). All analyses were conducted in SUDAAN V11.0.4 and accounted for the complex survey design of NHANES, including 8-year MEC weights.

## Figures and Tables

**Table 1:** Baseline demographics among **males**, and stratified by race/ethnicity, NHANES 2011–2018.

	Race/Ethnicity					
	Overall (N=5,938)	Non-Hispanic Whites (n=2,307)	Non-Hispanic Blacks (n=1,269)	Hispanics (n=1,364)	Non-Hispanic Asians (n=763)	Other (n=235)
Characteristic	% (SE)	% (SE)	% (SE)	% (SE)	% (SE)	% (SE)
Age* (years)	50.4 (0.3)	51.5 (0.3)	49.1 (0.4)	46.6 (0.4)	47.9 (0.5)	49.0 (1.1)
Age categories*, %								
30–44	36.1 (1.0)	32.2 (1.3)	38.7 (1.7)	49.8 (1.7)	44.0 (2.0)	43.1 (4.7)
45–54	24.1 (0.8)	23.9 (1.1)	26.5 (1.5)	23.8 (1.1)	25.5 (1.7)	21.2 (4.7)
55–64	24.1 (0.9)	25.9 (1.2)	23.0 (1.2)	17.7 (1.2)	19.8 (1.6)	20.7 (4.4)
65–75	15.7 (0.8)	18.0 (1.1)	11.8 (0.9)	8.8 (0.6)	10.7 (1.1)	15.0 (3.2)
Education*, %								
< HS education	13.7 (0.9)	8.6 (1.0)	18.5 (1.5)	37.3 (1.9)	13.3 (1.4)	12.8 (2.5)
HS graduate	22.7 (1.1)	22.3 (1.4)	29.2 (1.4)	24.5 (1.5)	12.0 (1.4)	20.7 (3.5)
Some college	29.1 (0.9)	30.5 (1.2)	31.6 (1.3)	22.7 (1.6)	15.1 (1.6)	38.5 (4.5)
College grad	34.5 (1.6)	38.6 (2.0)	20.7 (1.6)	15.5 (1.4)	59.6 (2.9)	28.0 (5.7)
Household Income*, %								
< $25K	15.3 (0.9)	11.8 (1.1)	27.2 (1.9)	25.9 (1.7)	10.4 (1.3)	19.1 (2.8)
$25K-<$55K	23.9 (1.0)	21.1 (1.2)	30.3 (1.6)	33.7 (1.5)	20.5 (1.8)	29.3 (4.2)
$55K-<$75K	13.5 (0.7)	13.4 (0.8)	12.5 (0.9)	14.3 (1.2)	12.1 (1.5)	15.4 (3.1)
≥ $75K	47.4 (1.7)	53.7 (2.0)	30.0 (2.3)	26.1 (1.8)	57.1 (2.9)	36.2 (5.6)
Nativity/years residing in the U.S.*, %								
U.S. Born	82.6 (1.0)	96.0 (0.5)	87.6 (1.4)	35.5 (2.5)	10.5 (1.4)	85.8 (3.8)
≥ 10 years in U.S.	14.1 (0.8)	3.1 (0.5)	9.5 (1.3)	54.4 (2.2)	68.1 (2.1)	11.9 (3.7)
< 10 years in U.S.	3.4 (0.4)	0.9 (0.3)	2.9 (0.5)	10.1 (1.3)	21.4 (2.1)	2.3 (1.2)
Has health insurance*, %	83.8 (0.9)	88.0 (1.1)	77.1 (1.6)	66.9 (1.6)	87.1 (1.4)	77.8 (3.2)
Married*, %	67.4 (1.2)	69.1 (1.5)	49.2 (1.7)	67.3 (1.3)	83.8 (1.3)	58.9 (5.0)
≥ 12 alcoholic drinks in past year*, %	77.9 (0.9)	81.0 (1.1)	71.0 (1.4)	77.2 (1.5)	57.1 (2.4)	66.9 (4.9)
Current smoker*, %	20.8 (0.7)	19.3 (0.9)	31.3 (1.4)	19.5 (1.4)	15.0 (1.5)	35.3 (4.5)
Physical activity level*, %								
None	44.3 (1.2)	42.6 (1.5)	47.7 (1.8)	51.2 (1.7)	40.4 (2.1)	47.6 (4.2)
Moderate	27.7 (1.2)	29.9 (1.6)	21.3 (1.1)	20.5 (1.4)	26.7 (2.0)	27.8 (4.1)
Vigorous	28.1 (1.3)	27.5 (1.7)	31.0 (1.6)	28.3 (2.0)	32.9 (2.0)	24.6 (4.2)
History of CVD*, %	9.4 (0.5)	10.0 (0.7)	10.0 (0.8)	5.8 (0.7)	5.3 (0.8)	16.0 (4.4)

**Abbreviations:** NHANES: National Health and Nutrition Examination Survey; CVD: Cardiovascular Disease; HS: High school

**Missing Data:** Alcohol 4.7%, History of CVD 0.2%, Insurance 0.1%

* Indicates significant differences by race/ethnicity (*P* <0.05)

**Table 2:** Baseline demographics among **females**, and stratified by race/ethnicity, NHANES 2011–2018.

	Race/Ethnicity					
	Overall (N=6,213)	Non-Hispanic Whites (n=2,290)	Non-Hispanic Blacks (n=1,367)	Hispanics (n=1,516)	Non-Hispanic Asians (n=841)	Other (n=199)
Characteristic	% (SE)	% (SE)	% (SE)	% (SE)	% (SE)	% (SE)
Age* (years)	51.0 (0.2)	52.3 (0.3)	49.0 (0.4)	47.3 (0.4)	49.2 (0.6)	48.3 (1.2)
Age categories*, %								
30–44	33.9 (0.9)	29.2 (1.2)	40.9 (1.7)	47.4 (1.8)	41.0 (2.2)	45.4 (5.5)
45–54	24.7 (0.9)	25.1 (1.2)	24.7 (1.2)	24.3 (1.2)	24.4 (1.8)	19.5 (3.9)
55–64	24.6 (0.7)	26.4 (1.0)	23.1 (1.5)	17.8 (0.9)	20.1 (1.3)	24.2 (3.9)
65–75	16.8 (0.6)	19.3 (0.8)	11.4 (0.7)	10.5 (0.7)	14.5 (1.7)	10.9 (2.6)
Education*, %								
< HS education	11.7 (0.8)	7.0 (0.9)	13.4 (1.0)	34.9 (2.1)	15.2 (1.8)	7.9 (2.7)
HS graduate	19.9 (0.7)	19.3 (1.0)	23.5 (1.2)	21.4 (1.1)	13.5 (1.4)	24.4 (5.3)
Some college	33.9 (1.0)	35.4 (1.4)	37.8 (1.4)	27.0 (1.6)	19.0 (1.5)	42.6 (5.0)
College grad	34.6 (1.6)	38.3 (2.0)	25.3 (1.5)	16.8 (1.4)	52.3 (2.2)	25.0 (4.7)
Household Income*, %								
< $25K	18.3 (0.9)	14.1 (1.2)	31.4 (1.6)	31.2 (1.7)	13.3 (1.7)	19.9 (3.0)
$25K-<$55K	26.6 (0.9)	24.5 (1.3)	33.9 (1.5)	31.2 (1.5)	22.8 (1.6)	36.1 (4.6)
$55K-<$75K	13.3 (0.7)	13.5 (0.8)	11.5 (1.1)	13.4 (0.9)	11.7 (1.6)	16.5 (4.2)
≥ $75K	41.8 (1.5)	47.8 (1.9)	23.2 (1.7)	24.3 (1.6)	52.2 (3.0)	27.6 (4.9)
Nativity/years residing in the U.S.*, %								
U.S. Born	82.6 (1.0)	95.9 (0.6)	88.9 (1.6)	38.1 (2.3)	7.7 (1.1)	83.8 (4.4)
≥ 10 years in U.S.	14.2 (0.8)	3.5 (0.5)	9.4 (1.5)	51.3 (2.0)	70.8 (1.7)	11.4 (3.7)
< 10 years in U.S.	3.3 (0.3)	0.6 (0.2)	1.6 (0.4)	10.6 (1.1)	21.5 (1.7)	4.8 (3.0)
Has health insurance*, %	87.5 (0.6)	91.2 (0.8)	82.9 (1.2)	69.8 (1.6)	88.9 (1.0)	90.3 (1.9)
Married*, %	59.8 (0.9)	63.4 (1.0)	33.9 (1.2)	56.5 (1.7)	77.4 (1.7)	51.1 (5.4)
≥ 12 alcoholic drinks in past year*, %	61.2 (1.3)	68.3 (1.6)	50.2 (1.7)	48.0 (1.9)	26.0 (1.5)	60.6 (4.9)
Current smoker*, %	16.9 (0.8)	18.1 (1.1)	19.4 (1.3)	10.5 (0.9)	2.3 (0.5)	34.4 (4.9)
Physical activity level*, %								
None	45.5 (1.2)	41.8 (1.5)	52.4 (2.0)	56.2 (1.6)	47.5 (2.0)	53.3 (4.9)
Moderate	32.4 (1.0)	34.5 (1.4)	28.4 (1.5)	25.3 (1.1)	34.5 (1.9)	27.2 (4.7)
Vigorous	22.1 (1.1)	23.7 (1.4)	19.2 (1.4)	18.5 (1.3)	18.0 (1.4)	19.5 (3.1)
History of CVD*, %	6.8 (0.4)	6.8 (0.6)	9.2 (0.9)	4.9 (0.6)	3.3 (0.7)	12.5 (3.2)

**Abbreviations:** NHANES: National Health and Nutrition Examination Survey; CVD: Cardiovascular Disease; HS: High school

**Missing Data:** Alcohol 7.8%, History of CVD 0.2%, Smoker 0.1%

* Indicates significant differences by race/ethnicity (*P* <0.05)

**Table 3.** Multivariable adjusted associations between SDoHs and Biological Age.

	Males (n=5,938)				Females (n=6,213)			
	Model 1	Model 2	Model 3	Model 4	Model 1	Model 2	Model 3	Model 4
	ß (95% CI)	ß (95% CI)	ß (95% CI)	ß (95% CI)	ß (95% CI)	ß (95% CI)	ß (95% CI)	ß (95% CI)
Education								
College graduate	-	-	-	-	-	-	-	-
Some college	1.33* (0.07, 2.60)	0.62 (−0.70,1.94)	−0.74 (−2.02, 0.54)	0.77 (−0.09, 1.62)	2.61* (1.25, 3.97)	1.88* (0.56, 3.20)	0.89 (−0.31, 2.09)	0.52* (0.08, 0.95)
HS graduate	1.35 (−0.16, 2.87)	0.61 (−0.88, 2.10)	−1.24 (−2.81, 0.33)	0.44 (−0.80, 1.68)	5.42* (4.26, 6.58)	4.58* (3.41, 5.74)	3.11* (2.00, 4.22)	0.80* (0.24, 1.35)
Less than HS	2.31* (0.75, 3.86)	1.49 (−0.29, 3.27)	−0.89 (−2.57,0.79)	0.17 (−1.04, 1.37)	5.14* (3.66, 6.61)	4.51* (3.07, 5.95)	3.06* (1.58, 4.54)	0.82* (0.10, 1.53)
Income								
≥ $75K	-	-	-	-	-	-	-	-
$55K-<$75K	2.50* (0.79, 4.20)	2.89* (1.12, 4.67)	1.75* (0.06, 3.44)	1.03 (−0.20, 2.26)	2.68* (1.20, 4.16)	2.19* (0.66, 3.72)	1.59* (0.14, 3.04)	0.43 (−0.21, 1.07)
$25K-<$55K	2.70* (1.39, 4.01)	4.64* (3.13, 6.15)	2.71* (1.21, 4.21)	0.80 (−0.03, 1.63)	3.59* (2.33, 4.84)	3.08* (1.79, 4.37)	2.05* (0.96, 3.14)	0.46 (−0.06, 0.98)
< $25K	4.13* (2.74, 5.51)	7.22* (5.59, 8.85)	4.95* (3.42, 6.48)	1.09 (−0.12, 2.30)	4.58* (3.17, 6.00)	4.01* (2.68, 5.35)	2.76* (1.51, 4.01)	0.66* (0.06, 1.27)
Nativity/years residing in the U.S.								
U.S. Born	-	-	-	-	-	-	-	-
≥ 10 years in the U.S.	−2.85* (−4.45, −1.25)	−2.68* (−4.09, −1.28)	−2.26* (−3.57, −0.95)	−2.46* (−3.54, −1.37)	−0.06 (−1.51, 1.39)	0.02 (−1.41, 1.44)	−0.53 (−1.85, 0.80)	−1.30* (−1.85, −0.74)
< 10 years in the U.S.	−9.53* (−11.35, −7.71)	−9.42* (−11.25, −7.58)	−7.97* (−9.65, −6.29)	−2.78* (−4.12, −1.44)	−6.39* (−8.73, −4.05)	−5.67* (−7.90, −3.44)	−6.11* (−8.09, −4.13)	−1.52* (−2.54, −0.51)

Model 1: adjusted for race/ethnicity

Model 2: adjusted for Model 1 variables and health insurance, marital status, education (in income and nativity models), income (in education and nativity models), and nativity (in education and income models)

Model 3: adjusted for Model 1 + Model 2 variables, and alcohol use, smoking history, physical activity, and history of CVD

Model 4: adjusted for Model 1 + Model 2 + Model 3 variables, and chronological age

* Significant at *P* < 0.05

## Data Availability

NHANES data are de-identified and publicly available through the National Center for Health Statistics and can be accessed via: https://wwwn.cdc.gov/nchs/nhanes/Default.aspx
